# Efficient, chemoselective synthesis of immunomicelles using single-domain antibodies with a C-terminal thioester

**DOI:** 10.1186/1472-6750-9-66

**Published:** 2009-07-20

**Authors:** Sanne WA Reulen, Ingrid van Baal, Jos MH Raats, Maarten Merkx

**Affiliations:** 1 Laboratory of Chemical Biology, Department of Biomedical Engineering, Eindhoven University of Technology, P.O. Box 513, 5600 MB Eindhoven, the Netherlands; 2 Department of Biomolecular Chemistry, Radboud University Nijmegen, P.O. Box 9101 6500 HB Nijmegen, the Netherlands; 3 ModiQuest B.V., Heijendaalseweg 135, 6525 AJ, Nijmegen, the Netherlands

## Abstract

**Background:**

Classical bioconjugation strategies for generating antibody-functionalized nanoparticles are non-specific and typically result in heterogeneous compounds that can be compromised in activity. Expression systems based on self-cleavable intein domains allow the generation of recombinant proteins with a C-terminal thioester, providing a unique handle for site-specific conjugation using native chemical ligation (NCL). However, current methods to generate antibody fragments with C-terminal thioesters require cumbersome refolding procedures, effectively preventing application of NCL for antibody-mediated targeting and molecular imaging.

**Results:**

Targeting to the periplasm of *E. coli *allowed efficient production of correctly-folded single-domain antibody (sdAb)-intein fusions proteins. On column purification and 2-mercapthoethanesulfonic acid (MESNA)-induced cleavage yielded single-domain antibodies with a reactive C-terminal MESNA thioester in good yields. These thioester-functionalized single-domain antibodies allowed synthesis of immunomicelles via native chemical ligation in a single step.

**Conclusion:**

A novel procedure was developed to obtain soluble, well-folded single-domain antibodies with reactive C-terminal thioesters in good yields. These proteins are promising building blocks for the chemoselective functionalization via NCL of a broad range of nanoparticle scaffolds, including micelles, liposomes and dendrimers.

## Background

The ability to raise antibodies with high affinity and specificity to almost any biomolecular target has made antibodies essential components in many biomedical fields, both in diagnostics and in the active targeting of drugs and contrast agents for molecular imaging [1]. For many of these applications there has been a drive to move towards smaller antibody formats, both to allow efficient recombinant production in *E. coli *and to potentially avoid unwanted immunogenic problems [2]. The ability to express these smaller antibody fragments in *E. coli *has also allowed the application of phage display approaches to allow in vitro screening of large libraries of antibody fragments. Nowadays, a wide range of smaller antibody formats are available including monovalent antibody fragments (Fab), single-chain antibody fragments (scFv), and single-domain antibodies (sdAb) [3]. The latter, which are sometimes also referred to as nanobodies, are derived from heavy-chain-only antibodies that have been found in camels, dromedaries, llamas and sharks [3,4]. Single-domain antibodies are the smallest antibody fragments available to date and have unique features including high solubility and thermal stability [4].

Current methods for bioconjugation of antibody fragments are non-specific and usually rely on amine and cysteine functionalities present on the protein surface [5]. This lack of control over the conjugation reaction gives rise to heterogeneous protein-nanoparticles. Moreover, the smaller size of single-domain antibodies compared to full size antibodies significantly increases the risk of affecting key residues near the antigen binding site when using non-specific conjugation strategies. In recent years several bioorthogonal ligation reactions that were originally developed in peptide chemistry have been applied for chemoselective protein functionalization of nanoparticles and chip surfaces [6-14]. Two examples of antibody conjugation using oxime chemistry were recently reported that take advantage of novel methods to selectively oxidize the N-terminus of antibodies or introduce genetically-encoded aldehyde tags at any position in the antibody sequence [15,16]. While promising, the applicability of oxime chemistry is still hampered by the incomplete introduction of ketone functionalities and the inability to use N-terminal acetylated proteins [17,18]. We and others have therefore explored the use of native chemical ligation (NCL) as an alternative chemoselective conjugation reaction, demonstrating its potential for the ligation of proteins to chip surfaces, dendrimers, supported lipid bilayers, micelles and liposomes [6,11,12,19-23]. Native chemical ligation is a chemoselective reaction under aqueous conditions between a C-terminal thioester and an N-terminal cysteine yielding a native peptide bond [24]. Site-specific coupling via NCL was made possible by the development of expression systems with self-cleavable intein domains to generate recombinant proteins with C-terminal thioesters [25].

Intein fusion proteins are normally expressed in the cytoplasm of *E. coli*, a reducing environment that prevents the proper formation of disulfide bonds that are essential for antibody stability. In vitro refolding of scFv-intein fusion proteins followed by on-column NCL has been reported [26], but the requirement to perform NCL on the column limits the applicability of this method. We recently reported a refolding procedure based on the redox couple sodium 2-mercaptoethanesulfonate (MESNA)/sodium 2,2-dithio-bis(ethanesulfonate) (diMESNA) to generate disulfide-containing proteins with a C-terminal MESNA thioester [27]. However, also for this method the requirement to do in-vitro refolding presents an important practical limitation. Here, we present an efficient strategy to obtain well-folded single-domain antibodies with a reactive C-terminal thioester by targeting the intein fusion protein to the periplasm of *E. coli*. Targeting antibody fragments to the oxidizing environment of the bacterial periplasm is known to increase the amount of active antibody fragments by allowing proper disulfide bond formation. Following this new procedure single-domain antibodies with C-terminal thioesters are obtained that can be directly coupled to cysteine-functionalized micelles to generate immunomicelles via native chemical ligation.

## Results and discussion

### Production of single-domain antibodies with a C-terminal thioester

**Figure 1:**
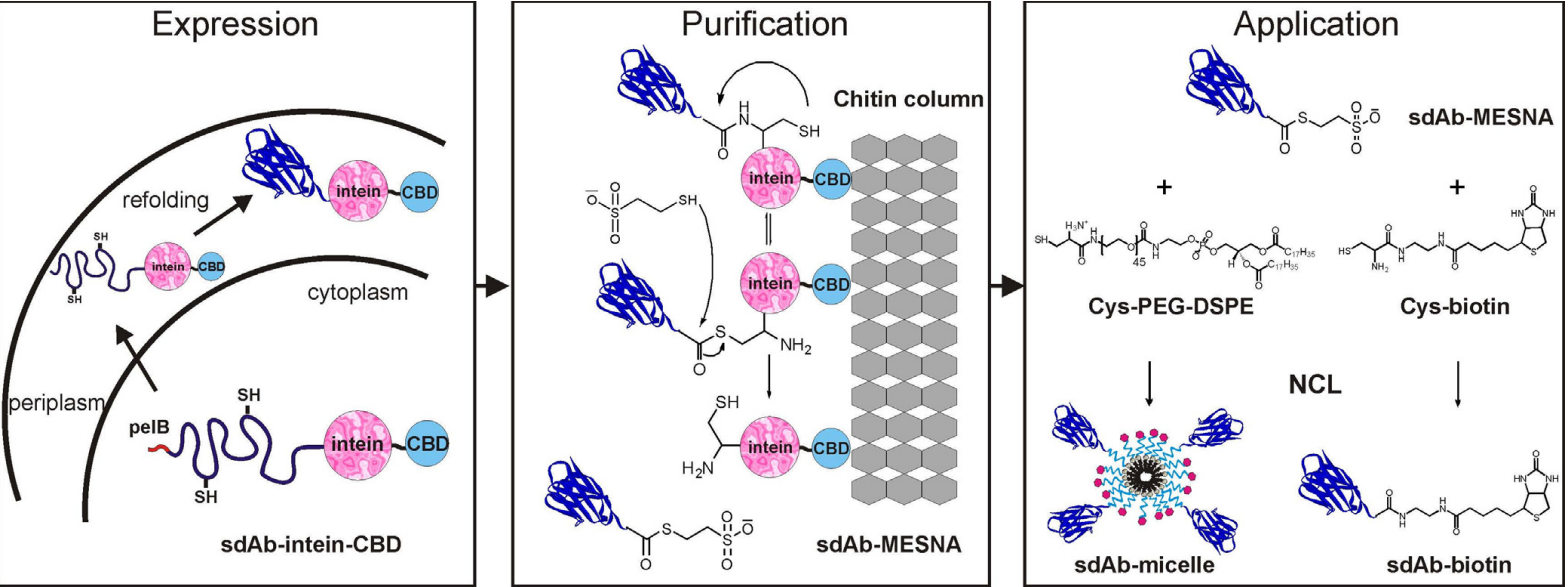
**Flow chart showing the approach explored in this work**. Introduction of a pelB leader sequence targets the sdAb-intein-CBD fusion protein to the periplasm, where the presence of disulfide isomerases and an oxidizing environment ensure proper folding and disulfide bond formation of the antibody domain. The chitin binding domain (CBD) allows single-step purification of the fusion protein from the periplasm, where after on-column treatment with MESNA results in intein-mediated cleavage and the formation of single-domain antibody with a reactive C-terminal thioester, which can then be directly applied in subsequent native chemical ligation reactions.

**Figure 2:**
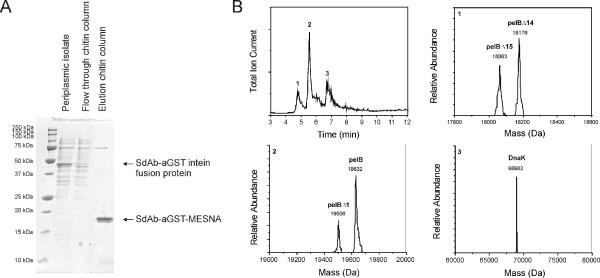
**Characterization of the single-domain antibody purification using SDS-PAGE and LC-MS**. (A) Expression and purification of the sdAb-aGST as intein fusion protein and subsequent generation of the C-terminal MESNA thioester via chitin affinity chromatography and intein mediated cleavage. (B) LC-MS analysis of the elution fraction of the chitin column. Top left panel: chromatogram; Top right and bottom panels: deconvoluted mass spectra of peaks 1, 2 and 3. (**pelB**: sdAb-aGST-MESNA with pelB leader attached (calcd. mass 19632 Da);**pelBΔ1**: sdAb-aGST-MESNA with the first amino acid of the pelB leader removed (calcd. mass 19501 Da); **pelBΔ14**: sdAb-aGST-MESNA with the first 14 amino acid of the pelB leader removed (calcd. mass 18175 Da); **pelBΔ15**: sdAb-aGST-MESNA with the first 15 amino acid of the pelB leader removed (calcd. mass 18062 Da); **DnaK **(calc. mass 68983 Da)).

Our approach, schematically depicted in Figure 1, was tested using a llama single-domain antibody obtained from screening a phage display library against glutathione-S from *Schistosoma japonicum*. The DNA sequence encoding for this antibody domain (sdAb-aGST) was provided in the pHENIX vector which contains an N-terminal sequence encoding a periplasmic leader sequence (pelB) and C-terminally a vesicular stomatitis virus (VSV-G) tag for detection purposes (see Additional file 1) [28]. The pelB leader sequence was used to target the protein to the oxidizing environment of the periplasm, because the sdAb-aGST protein contains a conserved disulfide bond that is known to be important for the stability of these single domain antibodies [29]. Since transport of the fusion protein to the periplasm could be dependent on the nature of the intein, we tested two commercially available intein expression vectors, pTXB1 and pTYB1 (NEB), that differ in the nature of the intein domain. The target gene was cloned at the N-terminus of the inteins while a chitin-binding domain (CBD) was present at the C-terminus of the intein for purification purposes. Both expression vectors were transformed into *E. coli *BL21 (DE3) and expression was assayed under a variety of conditions. SDS-PAGE analysis showed that expression using the pTXB1 vector allowed efficient transport of the intein fusion protein to the periplasm of *E. coli*, as evidenced by a band of 45 kDa in the periplasmic fraction (Figure 2). In contrast, expression of the single-domain antibody using the pTYB1 vector resulted in accumulation of the fusion protein as an insoluble aggregate in the cytoplasm of *E. coli *(not shown).

Figure 2 shows the purification of the MESNA thioester of sdAb-aGST (sdAb-aGST-MESNA) starting form the periplasmic fraction containing the sdAb-aGST-intein-CBD fusion protein. Upon loading the periplasmic fraction on a chitin column the sdAb-aGST-intein-CBD fusion protein binds to the chitin resin. The single-domain antibody with a C-terminal MESNA thioester was eluted from the chitin column after overnight incubation with the thiol MESNA. Yields for thioester terminated single-domain antibodies varied between 2 – 5 mg/L, which compares favorably with the 0.1–6 mg/L yields typically reported for single-domain antibodies via periplasmic expression in *E. coli *[30]. Since SDS-PAGE analysis showed the presence of two bands of slightly different molecular weight around 18 kDa, we performed LC-MS analysis to characterize the obtained proteins (Figure 2B). The main peak at 5.5 min contained sdAb-aGST-MESNA with the pelB leader attached (pelB). This peak also contained sdAb-aGST-MESNA with the first amino acid of the pelB leader removed (pelBΔ1). The minor peak at 5 min contained two sdAb-aGST-MESNA protein species with the first 14 and 15 amino acids of the pelB leader removed. The double band visible on the SDS-PAGE gel thus corresponds to multiple species of sdAb-aGST, all of which contain a C-terminal MESNA thioester. Addition of protease inhibitors during the purification procedure could not prevent this heterogeneous N-terminal processing. Similar incomplete pelB removal was observed before in *E. coli *BL21(DE3) strains and has been suggested to be due to the inability of its amino peptidase to efficiently cleave the pelB leader peptide [31]. The minor band observed on the SDS-PAGE gel around 70 kDa was identified using LC-MS as DnaK, a molecular chaperone protein that prevents aggregation of misfolded proteins in *E. coli*.

**Figure 3:**
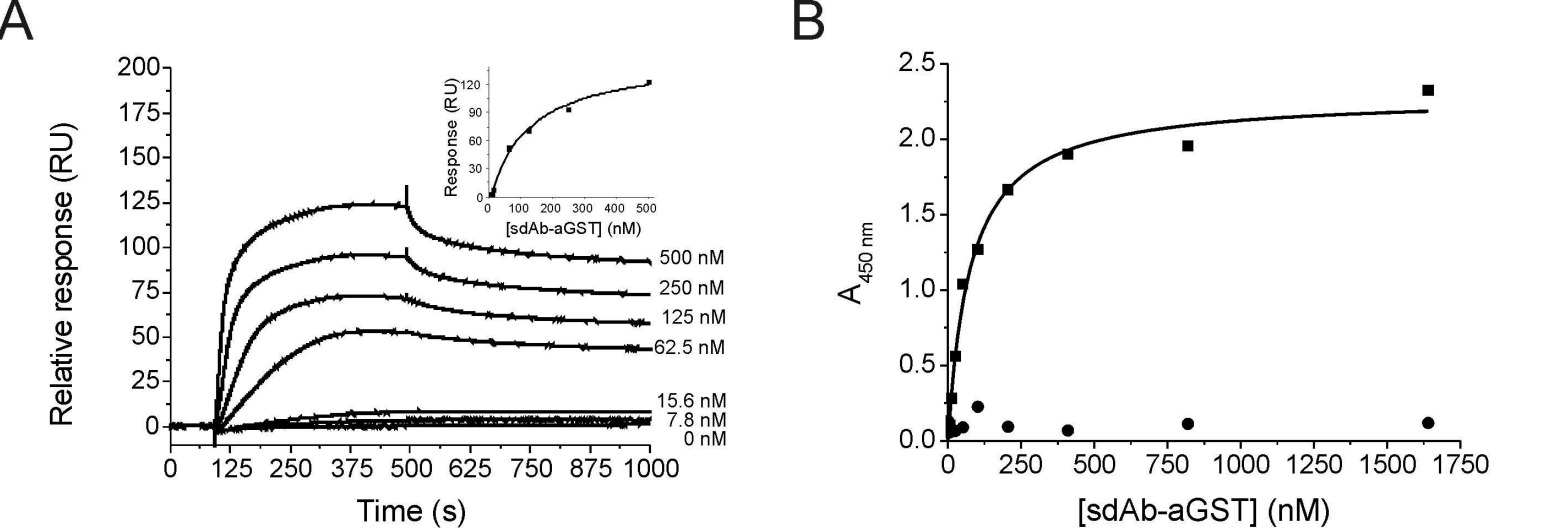
**The binding of the sdAb-aGST-MESNA to GST assayed with SPR and ELISA**. (A) SPR analysis of sdAb-aGST-MESNA binding to GST. Insert: steady state responses were plotted against the concentration. The line represents a fit to a one-site binding model using a *K*_*D *_of 116 nM. (B) ELISA measurement of binding of sdAb-aGST to GST (square) fitted with a one-site binding model using a *K*_D _of 77 nM and BSA (circle).

The sdAb-aGST protein contains a single disulfide bond that has previously been shown to play an essential role in the stability of these single-domain antibodies. To establish whether the reducing conditions used during MESNA cleavage affected the sdAb-aGST-MESNA produced in this manner, its interaction with GST was studied using Surface Plasmon Resonance (SPR). Initial attempts to immobilize the sdAb-aGST-MESNA to the CM5 Biacore chip using a standard amine coupling procedure resulted in a complete loss of GST binding capacity, suggesting that this classical amine coupling affects residues critical for antigen binding. The sdAb-aGST-MESNA protein contains 11 lysine residues, two of which are indeed located near the antigen binding site. To exclude antibody deactivation the assay was reversed and GST was immobilized on the CM5 chip. Figure 3A shows a clear binding response of the sdAb-aGST-MESNA to GST with an apparent dissociation constant of 120 nM. This number is in good agreement with results from an ELISA assay, which showed a dissociation constant of 80 nM (Figure 3B). The observed dissociation constants for the sdAb-aGST are similar to those reported for other single-domain antibodies [32]. In addition, SPR analysis of sdAb-aGST-MESNA after NCL with cysteine (sdAb-aGST-Cys) showed binding behavior that was similar to that of untreated sdAb-aGST (see Additional file 1). So while we cannot exclude at this time that the disulfide bond becomes transiently reduced during MESNA treatment or NCL, these procedures did not irreversibly affect the functionality of sdAb-GST.

### Preparation of immunomicelles with single-domain antibodies using NCL

**Figure 4:**
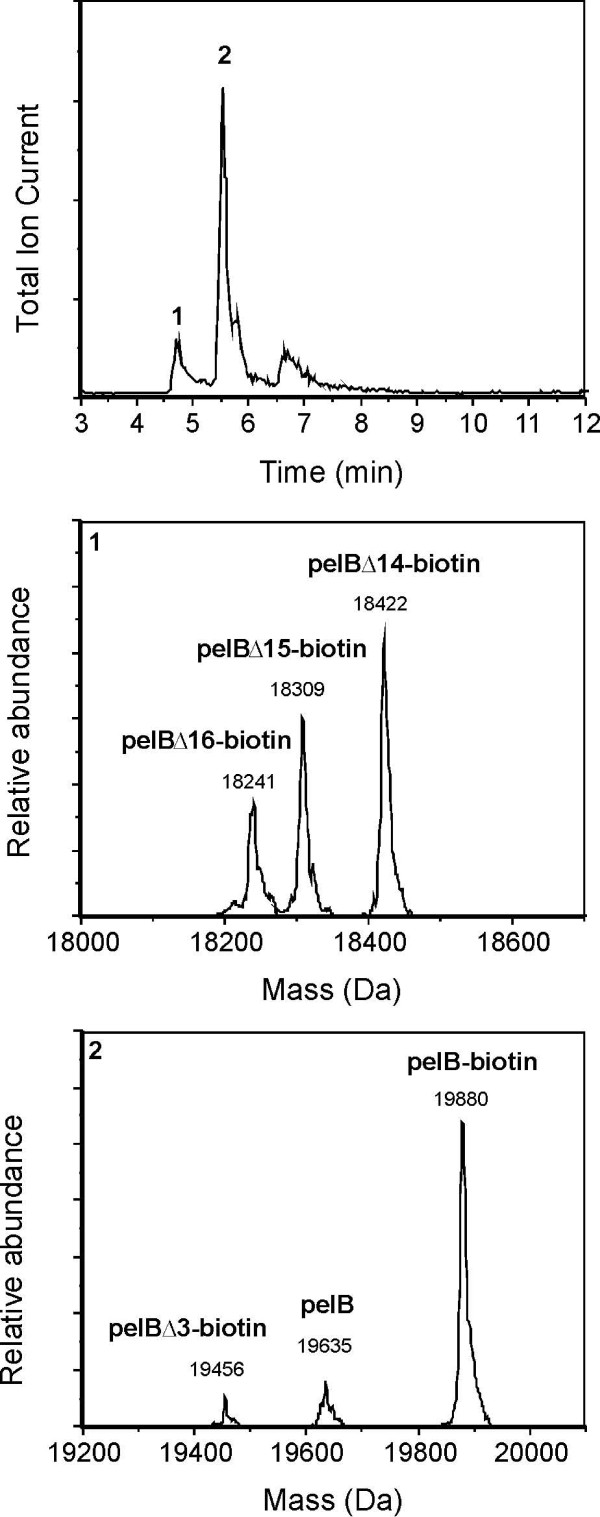
**Chromatogram and deconvoluted mass spectrum of the biotinylated single-domain antibody against GST after overnight ligation**. **pelB-biotin**: biotinylated sdAb-aGST with pelB leader attached (calcd. mass 19879 Da); **pelB**: MESNA thioester of the sdAb-aGST with pelB leader attached (calcd. mass 19632 Da); **pelBΔ3-biotin**: biotinylated sdAb-aGST with the first three amino acids of the pelB leader removed (calcd. mass 19457 Da);); **pelBΔ14-biotin**: biotinylated sdAb-aGST with the first 14 amino acids of the pelB leader removed (calcd. mass 18423 Da);**pelBΔ15-biotin**: biotinylated sdAb-aGST with the first 15 amino acids of the pelB leader removed (calcd. mass 18309 Da); **pelBΔ16-biotin**: biotinylated sdAb-aGST with the first 16 amino acids of the pelB leader removed (calcd. mass 18238 Da);

To test the performance of sdAb-aGST-MESNA in NCL reactions, we first assessed its reactivity towards cysteine-functionalized biotin. Ligation of this small molecule can be conveniently monitored using LC-MS. In addition, single-domain antibodies with C-terminal biotin groups are attractive for use in combination with the many streptavidin-modified compounds that are now commercially available. Figure 4 shows the LC-MS analysis of the product obtained after reaction of sdAb-aGST-MESNA with an excess of cysteine-functionalized biotin overnight at room temperature. Again two peaks appear in the chromatogram corresponding to sdAb-aGST with various fragments of the pelB leader sequence still attached. All peaks correspond to biotinylated forms of sdAb-aGST, with the exception of a minor peak at 19635 Da corresponding to a small amount of unreacted sdAb-aGST-MESNA.

**Figure 5:**
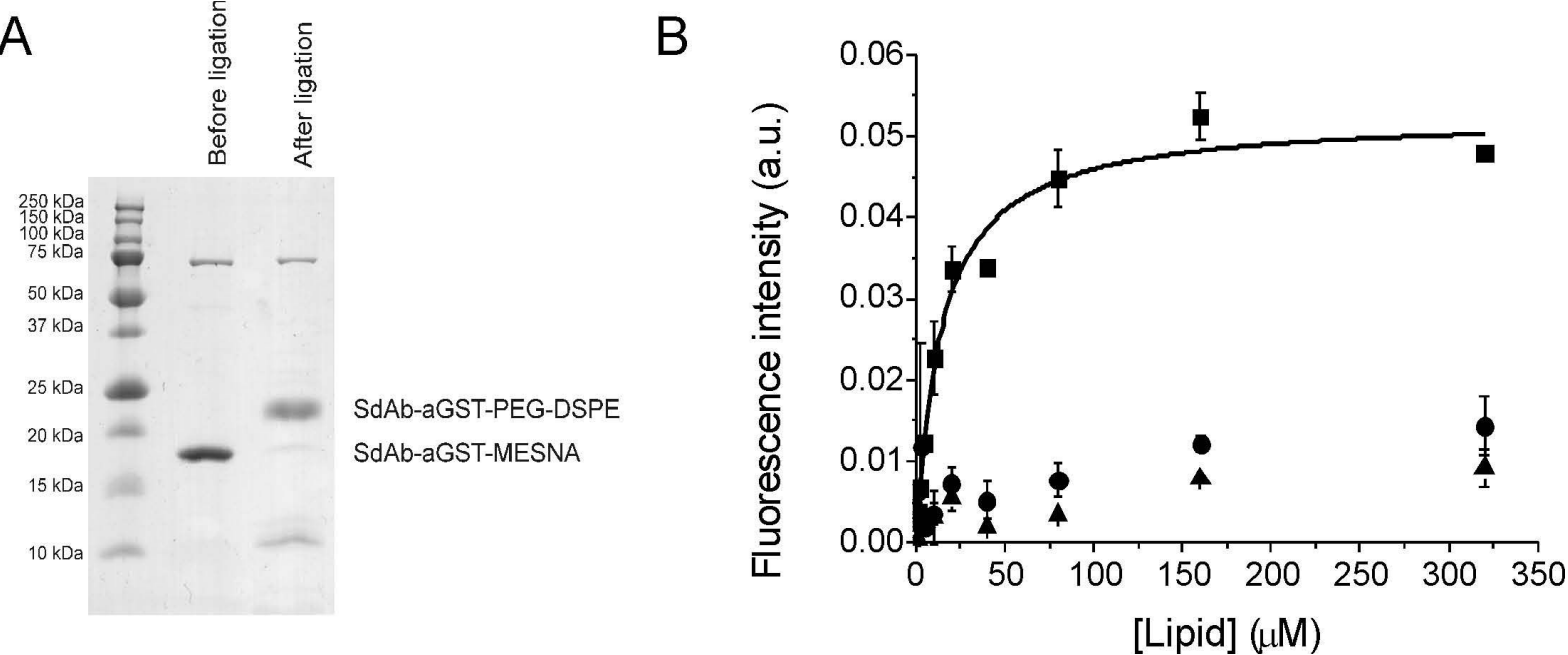
**Preparation and activity analysis of single-domain antibody micelles**. (A) SDS-PAGE analysis of NCL of 626 μM Cys-PEG-DSPE with 50 μM sdAb-aGST-MESNA. The reaction was performed overnight at room temperature in buffer containing 100 mM sodium phosphate, 50 mM MPAA, 10 mM TCEP, pH 6. (B) Solid-phase binding assay of sdAb-aGST functionalized micelles to GST (squares). Micelle binding was monitored by measuring the fluorescence of the rhodamine lipids at 620 nm using an excitation of 578 nm. Control experiments using non-modified micelles incubated on GST (triangles) and sdAb-aGST functionalized micelles on milk powder coated plates (diamonds) are also shown for comparison. The solid line represents a fit to a 1:1 binding model using an apparent *K*_*d *_of 14 μM.

Having established the essentially complete biotinylation of sdAb-aGST-MESNA, we next used the sdAb-aGST-MESNA to prepare immunomicelles [22,23]. Pegylated phospholipids such as PEG2000-DSPE are known to form relatively stable micelles with a critical micelle concentration (CMC) of ~5 μM, forming micelles with a diameter of 13 nm and approximately 90 lipids per micelle [33]. By attaching cysteine-functionalities at the end of the PEG-chain (Cys-PEG2000-DSPE) we recently showed that these micelles can be readily functionalized with up to 20 copies of the collagen binding protein CNA35 equipped with a C-terminal thioester. Here we used the same procedure to prepare micelles functionalized with sdAb-aGST. Micelles were prepared by mixing cysteine-functionalized phospholipids (Cys-PEG2000-DSPE) and rhodamine phospholipids in a 40:1 ratio, followed by addition of sdAb-aGST-MESNA in a 1:11 protein-lipid ratio. SDS-PAGE analysis showed essentially complete conversion to the lipidated form after overnight incubation with 50 mM 4-(carboxylmethyl) thiophenol (MPAA) (Figure 5A). Based on the aggregation number of 90 lipids per micelle and the full conversion of the sdAb-aGST to the lipidated form, one can calculate that the micelles contained on average eight sdAb-aGST proteins and two rhodamines. A solid-phase binding assay shows specific binding of the sdAb-aGST functionalized micelles to GST with half maximal binding at 14 μM PEG2000-DSPE. Since no binding was observed for non-functionalized micelles, and because the rhodamine-lipid is associated with the micelle via non-covalent interactions, this assay provides direct evidence for the specific binding of intact immunomicelles. It is important to note that the apparent dissociation constant of ~14 μM is based on the lipid concentration and reflects the stability of the micelles and not the intrinsic affinity of the sdAb domains attached to the micelles. A similar phenomenon was observed previously for the binding of CNA35-functionalized micelles to collagen, which also showed half maximal binding at a lipid concentration of ~10 μM, despite the fact that CNA35 by itself binds collagen with 0.5 μM affinity [23].

## Conclusion

An efficient strategy was developed that generates active, folded single-domain antibodies with a reactive C-terminal thioester for direct application in nanoparticle functionalization via native chemical ligation. Two applications were reported here to illustrate their potential as generic building blocks for the generation of targeted nanoparticles, the generation of biotinylated single-domain antibodies and the efficient preparation of immunomicelles. Finally, the approach of targeting intein fusion proteins to the bacterial periplasm may provide a generic, attractive alternative to existing methods to obtain disulfide-containing proteins with a C-terminal thioester.

## Methods

### General

Unless stated otherwise, all reagents and chemicals were obtained from commercial sources and used without further purification. Cysteine-functionalized 1,2-Distearoyl-sn-glycero-3-phosphoethanolamine-*N*-[amino(poly(ethylene glycol))2000] (Cys-PEG-DSPE) was prepared according to a literature procedure [22]. The expression vectors pTXB1 and pTYB1, restriction enzymes and chitin beads were purchased from New England Biolabs (Beverly, MA). Competent cells were purchased from Novagen (Darmstadt, Germany). Monoclonal mouse anti-vsv glycoprotein antibody (V5507) was purchased from Sigma (St. Louis, MO). HRP-conjugated rabbit anti-mouse immunoglobulin polyclonal antibody (P0260) was purchased from DakoCytomation (Heverlee, The Netherlands). UV-Vis spectra were recorded on a Shimadzu Multispec 1501 spectrophotometer. Primers used for all the cloning procedures were supplied by MWG (Ebersberg, Germany).

### Plasmid constructs

The GST-specific single-domain antibody was obtained from a naïve llama-derived nanobody library described previously [34]. Selection of phages for binding to bacterial expressed recombinant GST (pGEX-4T-2 vector) was performed essentially as described in [35,36]. The gene encoding for sdAb-aGST was amplified by PCR from vector pHENIX sdAb-α GST C11 with the primer pair pUC119pelB_f (5'-GGTGGTCATATGAAATACCTATTGCCTACGGCAGC-3') and pUC119vsv_r (5'-GGTGGTTGCTCTTCCGCATGCGGCCCCCTTTCCAAG-3'). The PCR products and the pTXB1 and pTYB1 vectors were double-digested with the restriction endonucleases *Nde *I and *Sap *I followed by ligation of the amplified DNA fragments in the open plasmids yielding the expression plasmids pTXB1-sdAb-aGST and pTYB1-sdAb-aGST. DNA sequencing (BaseClear, Leiden, The Netherlands) using T7 promoter and intein-specific reversed primers (New England Biolabs) confirmed the correct in-frame fusion of the proteins with the intein sequence.

### Protein Expression and Purification

The expression plasmids were transformed into *E. coli *BL21 (DE3) cells. Expression experiments were performed in 0.5 L LB cultures supplemented with 100 μg/mL ampicillin by inoculating with a 5 mL overnight culture. The cultures were grown at 37°C and 250 rpm until the OD_600 _reached a value between 0.5–0.6. Protein expression was induced with 0.2 mM IPTG and cultures were incubated for 3 hours at 30°C. Cells were harvested by centrifugation for 10 min at 8,000 g at 4°C, after which the medium was discarded and the cell pellets were resuspended in 85 mL TES (30 mM Tris-HCl pH 8.0, 1 mM EDTA, 20% (w/v) sucrose) and incubated at room temperature for 10 minutes with continuous shaking. After centrifugation for 10 min at 8,000 g and 4°C, the pellet was resuspended in 85 mL of ice-cold 5 mM MgSO_4 _and incubated on ice for 10 minutes with occasional shaking. After centrifugation for 10 min at 8,000 g at 4°C, 2 mL 0.5 M Tris-HCl pH 7.5 was added to the supernatant. ATP (3 mM) was added to the supernatant and incubated for 1–3 hours at 4°C to suppress binding of DnaK to the sdAb-aGST intein fusion protein. The periplasmic protein fraction was directly applied to a chitin column that was equilibrated with 10 column volumes of column buffer (20 mM sodium phosphate, 0.1 mM EDTA, 0.5 M NaCl, 3 mM ATP, pH 8). The column was washed with 10 volumes of column buffer after which the columns were quickly flushed with 3 column volumes of cleavage buffer (20 mM sodium phosphate, 0.1 mM EDTA, 0.5 M NaCl, 100 mM MESNA, pH 6) and incubated overnight at 4°C. Proteins were eluted in cleavage buffer without MESNA and pooled. The protein concentration was determined by UV-Vis using ε_280 nm _= 24,535 M^-1^cm^-1 ^or using a Quant-it protein assay (Invitrogen) according to the manufacturers instructions.

### LC-MS

Samples for LC-MS analysis were concentrated with Biomax centrifugation filters (MWCO 5 kDa) for small volumes and Amicon centrifugational filters (MWCO 10 kDa) for large volumes. Samples were buffer exchanged to a mixture of 9:1 H_2_O/acetonitrile or to 50 mM ammonium acetate pH 6.8. Reversed phase HPLC was performed on a Vydac protein column with a mobile phase of H_2_O/acetonitrile with 0.1% TFA. ESI-MS spectra were measured on a Thermo Finnigan LCQ Deca XP MAX in positive mode. MagTran software was used for deconvolution.

### ELISA

All washing steps were performed with 200 μl MPBST (5% milk powder, 0.05% Tween20 in PBS (137 mM NaCl, 2.7 mM KCl, 4.3 mM Na_2_HPO_4_, 1.4 mM KH_2_PO_4_, pH 7.4)) unless stated otherwise. 96 wells Corning EIA/RIA Microplates were coated overnight at 4°C with 0.5 μg/well (50 μl) GST or BSA in 0.1 M NaHCO_3 _pH 8.6. After overnight incubation the plates were washed 3 times with PBS/0.05% Tween20. Plates were blocked with 50 μl MPBST for 1 hour at room temperature. After washing the plates 3 times, 50 μl samples of sdAb-aGST-MESNA were loaded and incubated for 1 hour at room temperature. Plates were washed 6 times and subsequently incubated with 50 μl mouse Anti-VSV monoclonal antibody (5000× diluted) for 1 hour. Plates were washed 6 times and subsequently incubated with 50 μl alkaline HRP-conjugated rabbit anti-mouse immunoglobulin polyclonal antibody (2500× diluted), for 1 hour. Plates were washed 6 times and subsequently incubated with 50 μl/well detection solution (1:1 peroxide buffer (UP)/Tetramethylbenzidine (TMB), BioMérieux, Boxtel, The Netherlands). Reaction was stopped after 5 to 10 min with 50 μl 2 M sulfuric acid. The absorbance was measured at 450 nm in duplicate on a Thermo multiskan ascent platereader.

### Solid phase binding assay with immunomicelles

All washing steps were performed with 200 μl HBS. 96 wells Corning EIA/RIA Microplates were coated overnight at 4°C with 0.5 μg/well (50 μl) GST or milk powder in HBS (10 mM HEPES, 135 mM NaCl, pH 7.4). After overnight incubation the plates were washed 3 times. Plates were blocked with 50 μl MHBS (5% milk powder in HBS) for 3 hours at room temperature. After washing the plates 3 times, 50 μl samples of sdAb-aGST micelles were loaded and incubated for 4 hours at room temperature. Plates were washed 8 times after which the fluorescence of the rhodamine-containing micelles was measured in duplicate on a Thermo Fluoroskan Ascent FL plate reader (excitation at 578 nm, emission at 620 nm).

### Surface Plasmon Resonance

Sensorgrams were obtained on a Biacore T100 (GE Healthcare) using a CM5 chip functionalized with GST using standard EDC/NHS protocols. All binding experiments were performed at 25°C using PBS with 0.05% Tween-20 (PBST) as running buffer at a flow rate of 30 μL/min. Samples containing 0–500 nM of sdAb-aGST-MESNA in PBST were injected on a CM5 chip functionalized with GST (160 RU immobilized) for 400 sec, followed by a 10 min dissociation phase. Regeneration of the chip was performed with a 30 s injection of 10 mM NaOH. Aspecific binding and buffer effects were taken into account by subtracting the simultaneous response from a reference surface functionalized with ethanolamine.

### Ligation of cysteine-functionalized biotin to sdAb-aGST

Cysteine-functionalized biotin was prepared according to a literature procedure and checked with NMR and LC-MS analysis [37]. A reaction mixture was prepared containing 7 μM sdAb-α GST-MESNA in 20 mM sodium phosphate, 0.1 mM EDTA, 0.5 M NaCl, pH 6 to which 1 mM cysteine-functionalized biotin, 50 mM MPAA and 10 mM tris(2-carboxyethyl) phosphine hydrochloride (TCEP) were added. The final pH of the reaction mixture was 5.5. The reaction was incubated overnight at 20°C and analyzed using LC-MS analysis.

### Ligation of sdAb-αGST to cysteine-functionalized micelles

A mixture of 97.5 mol percent Cys-PEG-DSPE and 2.5 mol percent rhodamine-DPPE in chloroform was placed in a vial. After chloroform evaporation the obtained lipid film was rehydrated in 0.1 M sodium phosphate pH 8.5 ([lipid] = 640 μM) with 50 mM MPAA and 10 mM TCEP, vortexed for 2 min followed by 5 min sonication. SdAb-aGST-MESNA was added to a final concentration of 50 μM (final pH was 6.7). After overnight incubation at room temperature the protein micelles were analyzed using SDS-PAGE to check for complete conversion to the lipidated protein.

## Authors' contributions

SWAR developed the new expression system for antibody fragments and wrote the manuscript. IvB synthesized cysteine-functionalized biotin and supervised part of the research. JMHR provided the pHENIX plasmid with the sdAb-aGST and helped with the experimental procedures concerning the single-domain antibodies. MM was responsible for the conceptual design of this project and contributed to the writing of the manuscript. All authors read and approved the final manuscript.

## Supplementary Material

Additional file 1 **Supporting information**. The binding of sdAb-aGST functionalized with cysteine to GST assayed using SPR (figure S1), and the DNA and protein sequence of the sdAb-aGST. Click here for file

## References

[B1] Wu AM, Senter PD (2005). Arming antibodies: prospects and challenges for immunoconjugates. Nat Biotechnol.

[B2] Fernandez LA (2004). Prokaryotic expression of antibodies and affibodies. Curr Opin Biotechnol.

[B3] Holliger P, Hudson PJ (2005). Engineered antibody fragments and the rise of single domains. Nat Biotechnol.

[B4] Revets H, De Baetselier P, Muyldermans S (2005). Nanobodies as novel agents for cancer therapy. Expert Opin Biol Ther.

[B5] Nobs L, Buchegger F, Gurny R, Allemann E (2004). Current methods for attaching targeting ligands to liposomes and nanoparticles. J Pharm Sci.

[B6] Camarero JA, Kwon Y, Coleman MA (2004). Chemoselective attachment of biologically active proteins to surfaces by expressed protein ligation and its application for "protein chip" fabrication. J Am Chem Soc.

[B7] Kalia J, Abbott NL, Raines RT (2007). General method for site-specific protein immobilization by staudinger ligation. Bioconjug Chem.

[B8] Jonkheijm P, Weinrich D, Schröder H, Niemeyer CM, Waldmann H (2008). Chemical strategies for generating protein biochips. Angew Chem Int Ed Engl.

[B9] Lempens EH, Helms BA, Merkx M, Meijer EW (2009). Efficient and chemoselective surface immobilization of proteins by using aniline-catalyzed oxime chemistry. ChemBioChem.

[B10] Camarero JA (2008). Recent developments in the site-specific immobilization of proteins onto solid supports. Biopolymers.

[B11] Kwon Y, Coleman MA, Camarero JA (2006). Selective immobilization of proteins onto solid supports through split-intein-mediated protein trans-splicing. Angew Chem Int Ed Engl.

[B12] Helms B, Van Baal I, Merkx M, Meijer EW (2007). Site-specific protein and peptide immobilization on a biosensor surface by pulsed native chemical ligation. ChemBioChem.

[B13] Watzke A, Kohn M, Gutierrez-Rodriguez M, Wacker R, Schroder H, Breinbauer R, Kuhlmann J, Alexandrov K, Niemeyer CM, Goody RS (2006). Site-selective protein immobilization by staudinger ligation. Angew Chem Int Ed Engl.

[B14] Dirksen A, Dawson PE (2008). Expanding the scope of chemoselective peptide ligations in chemical biology. Curr Opin Chem Biol.

[B15] Scheck RA, Francis MB (2007). Regioselective labeling of antibodies through N-terminal transamination. ACS Chem Biol.

[B16] Wu P, Shui W, Carlson BL, Hu N, Rabuka D, Lee J, Bertozzi CR (2009). Site-specific chemical modification of recombinant proteins produced in mammalian cells by using the genetically encoded aldehyde tag. Proc Natl Acad Sci USA.

[B17] Scheck RA, Dedeo MT, Iavarone AT, Francis MB (2008). Optimization of a biomimetic transamination reaction. J Am Chem Soc.

[B18] Rush JS, Bertozzi CR (2008). New aldehyde tag sequences identified by screening formylglycine generating enzymes in vitro and in vivo. J Am Chem Soc.

[B19] Olschewski D, Becker CFW (2008). Chemical synthesis and semisynthesis of membrane proteins. Mol BioSyst.

[B20] van Baal I, Malda H, Synowsky SA, van Dongen JL, Hackeng TM, Merkx M, Meijer EW (2005). Multivalent peptide and protein dendrimers using native chemical ligation. Angew Chem Int Ed Engl.

[B21] Grogan MJ, Kaizuka Y, Conrad RM, Groves JT, Bertozzi CR (2005). Synthesis of lipidated green fluorescent protein and its incorporation in supported lipid bilayers. J Am Chem Soc.

[B22] Reulen SW, Brusselaars WW, Langereis S, Mulder WJ, Breurken M, Merkx M (2007). Protein-liposome conjugates using cysteine-lipids and native chemical ligation. Bioconjug Chem.

[B23] Reulen SWA, Dankers PYW, Bomans PHH, Meijer EW, Merkx M (2009). Collagen targeting using protein-functionalized micelles: the strength of multiple weak interactions. J Am Chem Soc.

[B24] Dawson PE, Muir TW, Clark-Lewis I, Kent SB (1994). Synthesis of proteins by native chemical ligation. Science.

[B25] Muir TW, Sondhi D, Cole PA (1998). Expressed protein ligation: a general method for protein engineering. Proc Natl Acad Sci USA.

[B26] Sydor JR, Mariano M, Sideris S, Nock S (2002). Establishment of intein-mediated protein ligation under denaturing conditions: C-terminal labeling of a single-chain antibody for biochip screening. Bioconjugate Chem.

[B27] Bastings MMC, van Baal I, Meijer EW, Merkx M (2008). One-step refolding and purification of disulfide-containing proteins with a C-terminal MESNA thioester. BMC Biotechnol.

[B28] Raats J, van Bree N, van Woezik J, Pruijn G (2003). Generating recombinant anti-idiotypic antibodies for the detection of haptens in solution. J Immunoassay Immunochem.

[B29] Hagihara Y, Matsuda T, Yumoto N (2005). Cellular quality control screening to identify amino acid pairs for substituting the disulfide bonds in immunoglobulin fold domains. J Biol Chem.

[B30] Saerens D, Pellis M, Loris R, Pardon E, Dumoulin M, Matagne A, Wyns L, Muyldermans S, Conrath K (2005). Identification of a universal VHH framework to graft non-canonical antigen-binding loops of camel single-domain antibodies. J Mol Biol.

[B31] Bruser T, Deutzmann R, Dahl C (1998). Evidence against the double-arginine motif as the only determinant for protein translocation by a novel Sec-independent pathway in Escherichia coli. FEMS Microbiol Lett.

[B32] Saerens D, Kinne J, Bosmans E, Wernery U, Muyldermans S, Conrath K (2004). Single domain antibodies derived from dromedary lymph node and peripheral blood lymphocytes sensing conformational variants of prostate-specific antigen. J Biol Chem.

[B33] Ashok B, Arleth L, Hjelm RP, Rubinstein I, Onyuksel H (2004). In vitro characterization of PEGylated phospholipid micelles for improved drug solubilization: effects of PEG chain length and PC incorporation. J Pharm Sci.

[B34] Roodink I, Raats J, Zwaag B van der, Verrijp K, Kusters B, van Bokhoven H, Linkels M, de Waal RMW, Leenders WPJ (2005). Plexin D1 expression is induced on tumor vasculature and tumor cells: A novel target for diagnosis and therapy?. Cancer Res.

[B35] Hof D, Hoeke MO, Raats JM (2008). Multiple-antigen immunization of chickens facilitates the generation of recombinant antibodies to autoantigens. Clin Exp Immunol.

[B36] Raats JH, Wijnen EM, Pruijn GM, van den Hoogen FH, van Venrooij WJ (2003). Recombinant human monoclonal autoantibodies specific for citrulline-containing peptides from phage display libraries derived from patients with rheumatoid arthritis. J Rheumatol.

[B37] Tolbert TJ, Wong C-H (2000). Intein-mediated synthesis of proteins containing carbohydrates and other molecular probes. J Am Chem Soc.

