# Supplementary material for: Efficient, chemoselective synthesis of immunomicelles using single-domain antibodies with a C-terminal thioester
Source: BMC Biotechnol. 2009 Jul 20;9:66. doi: 10.1186/1472-6750-9-66 (PMC2719619; doi:10.1186/1472-6750-9-66)
Supplement: Additional file 1 — Supporting information. The binding of sdAb-aGST functionalized with cysteine to GST assayed using SPR (figure S1), and the DNA and protein sequence of the sdAb-aGST. [file 1472-6750-9-66-S1.pdf]

Supporting information

Efficient, chemoselective synthesis of immunomicelles using single-domain antibodies with a C-terminal thioester

Sanne W.A. Reulen, Ingrid van Baal, Jos M.H. Raats, and Maarten Merkx

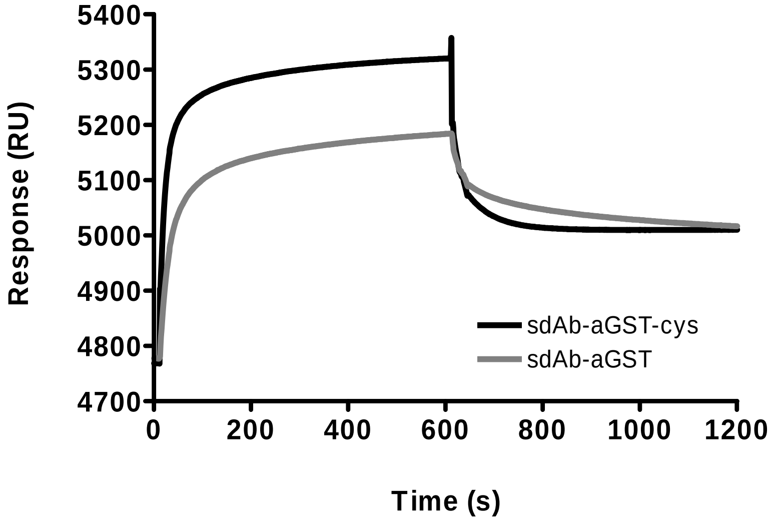

Figure S1: *The binding of cysteine functionalized sdAb-αGST (sdAb-aGST-cys, black) and sdAb-αGST with a carboxy termini (sdAb-aGST, grey) to GST assayed with SPR. SdAb-aGST-cys was injected at a concentration of 800 nM in PBS supplemented with 0.1 % Tween-20 (PBST) on an CM5 chip functionalized with gluthation-S transferase (2000 RU bound) for 10 min followed by a 10 min dissociation phase. For comparison sdAb-aGST was injected at a concentration of 500 nM in PBST on the same chip.*

DNA sequence sdAb-aGST:

ATGAAATACCTATTGCCTACGGCAGCCGCTGGATTGTTATTACTCGCGGCCCAGCCGGCC

ATGGCCCAGGTGCAGCTGCAGGACTCCGGGGGAGGCATCGTGCAGCCTGGGGGTTCTCTG

AGACTCTCCTGTGCAGGCTCTGGATTCAGTTTGGATCATTATGCCATAGGCTGGTTCCGC

CAGGCTCCAGGGAAGCGGCGCGAGTTGGTCGCAAGTATAACTAGTGGTGGTACCATAAGG

TATGCAGACTCCGTGAAGGACCGATTCACCATCTCCAGAGACAACGCCAAGAACAGGGTG

TATCTGCAAATGAACAGCCTGAAACCTGAAGACACGGCCGTGTATTACTGTTCAGCTCGC

GCCAAATTTAGTACTTCCTATGACTACTGGGGCCAGGGGACCCAGGTCACCGTCTCCTCG

GAACCCAAGACACCAAAACCCAAGACACCAAAACCACAACCAGCGGCCGCACATCATCAC

CATCATCACCATCATTATACAGACATAGAGATGAACCGACTTGGAAAGGGGGCCGCA

Protein sequence sdAb-aGST:

pelB framework-1
M K Y L L P T A A A G L L L L A A Q P A M A Q V Q L Q D S G

cdr1
G G I V Q P G G S L R L S C A G S G F S L D H Y A I G W F R

framework-2 cdr2
Q A P G K R R E L V A S I T S G G T I R Y A D S V K D R F T

framework-3
I S R D N A K N R V Y L Q M N S L K P E D T A V Y Y C S A R

cdr3 framework-4
A K F S T S Y D Y W G Q G T Q V T V S S E P K T P K P K T P

His-tag vsv-tag
K P Q P A A A H H H H H H H H Y T D I E M N R L G K G A A
